# Robot-Assisted Therapy and Constraint-Induced Movement Therapy for Motor Recovery in Stroke: Results From a Randomized Clinical Trial

**DOI:** 10.3389/fnbot.2021.684019

**Published:** 2021-07-21

**Authors:** Thais Tavares Terranova, Marcel Simis, Artur César Aquino Santos, Fábio Marcon Alfieri, Marta Imamura, Felipe Fregni, Linamara Rizzo Battistella

**Affiliations:** ^1^Instituto de Medicina Fisica e Reabilitacao, Hospital das Clinicas da Faculdade de Medicina da Universidade de Sao Paulo, Sao Paulo, Brazil; ^2^Physical Medicine and Rehabilitation, Harvard Medical School, Boston, MA, United States; ^3^Physiatry, Faculdade de Medicina da Universidade de Sao Paulo, Sao Paulo, Brazil

**Keywords:** stroke, robot-assisted therapy, constraint-induced movement therapy, exoskeleton, motor recovery

## Abstract

**Background:** Stroke is one of the leading causes of adult disability, and up to 80% of stroke survivors undergo upper extremity motor dysfunction. Constraint-Induced Movement Therapy (CIMT) and Robot-Assisted Therapy (RT) are used for upper limb stroke rehabilitation. Although CIMT and RT are different techniques, both are beneficial; however, their results must be compared. The objective is to establish the difference between RT and CIMT after a rehabilitation program for chronic stroke patients.

**Method:** This is a randomized clinical trial, registered at ClinicalTrials.gov (ID number NCT02700061), in which patients with stroke received sessions of RT or CIMT protocol, combined with a conventional rehabilitation program for 12 weeks. The primary outcome was measured by Wolf Motor Function Test (WMFT) and Fugl-Meyer Assessment—Upper Limb (FMA-UL). Activities of daily living were also assessed.

**Results:** Fifty one patients with mild to moderate upper limb impairment were enrolled in this trial, 25 women and 26 men, mean age of 60,02 years old (SD 14,48), with 6 to 36 months after stroke onset. Function significantly improved regardless of the treatment group. However, no statistical difference was found between both groups as *p*-values of the median change of function measured by WMFT and FMA were 0.293 and 0.187, respectively.

**Conclusion:** This study showed that Robotic Therapy (RT) was not different from Constraint-Induced Movement Therapy (CIMT) regardless of the analyzed variables. There was an overall upper limb function, motor recovery, functionality, and activities of daily living improvement regardless of the interventions. At last, the combination of both techniques should be considered in future studies.

## Introduction

Stroke is one of the leading causes of disability worldwide. Up to 80% of stroke survivors will endure upper limb motor dysfunction with reduced ability to perform daily living activities, an important issue of public health (Nichols-Larsen et al., 2005; Levin et al., 2009; Langhorne et al., 2011; Johnson et al., 2016). Among the techniques focused on upper limb motor recovery, two have gained prominence, Constraint-Induced Movement Therapy (CIMT) and Robot-Assisted Therapy (RT).

RT is based on the concept of high intensity and an increased number of repetitions of functional movements to induce upper limb functional improvement (Krebs and Hogan, 2006; Dipietro et al., 2012) and has been reported as a potential approach to provide stroke patients with motor and functional recovery of upper limbs, presenting promising results in the literature (Ferraro et al., 2003; Hesse et al., 2003; Dipietro et al., 2005; Krebs and Hogan, 2006; Krebs et al., 2008; Volpe et al., 2008; Lo et al., 2010).

Robotic devices provide stroke patients with intensive stimuli and allow the professionals to control the parameters during the rehabilitation session (Ferraro et al., 2003; Hesse et al., 2003; Dipietro et al., 2005; Krebs and Hogan, 2006; Krebs et al., 2008; Volpe et al., 2008; Lo et al., 2010). These devices may include the combination of upper limb functional movements within three modalities: passive, active-assisted, or active-resisted (Ferraro et al., 2003; Dipietro et al., 2005). Robotic devices may be advantageous when compared to traditional rehabilitation programs, such as the output of objective measures, e.g., speed, torque, range of motion, position, and others, that evaluate and monitor the patient evolution, and the customization of treatment sessions concerning different levels of movement impairment (Lo et al., 2010). Also, the consistency and reproducibility of robotic-aided training allow RT in multicentric clinical trials (Hesse et al., 2003).

The Constraint-Induced movement therapy (CIMT) aims to improve motor function of the paretic limb by combining an intensive training program with the restraint of the unaffected arm (Wolf et al., 2002). This approach includes shaping procedures and task practice during the upper limb restriction for 90% of the day. CIMT also has a subjective behavioral contract that must be established between the patient and the therapist, especially in the performance in the home activities (Morris et al., 2006; Taub et al., 2006). The restriction of the unaffected arm marks CIMT during the treatment and intensive training of the affected upper limb, including behavioral techniques (Thrane et al., 2014). This technique is currently considered the gold-standard intervention for treating the paretic upper limb (Wolf et al., 1989, 2002; Taub et al., 1993, 2006; Kunkel et al., 1999; Barreca et al., 2003; Dettmers et al., 2005; Morris et al., 2006; Page and Levine, 2007).

A review that compared CIMT with conventional therapy and no treatment showed improvements of upper limb motor function represented by standardized mean difference (SMD) 0.34 (95% CI 0.12 to 0.55), which demonstrates a significant difference in favor of CIMT (*p* = 0.004). Nonetheless, this review had limited power due to small sample sizes and weak study designs (Corbetta et al., 2015). Regarding RT, a systematic review showed that robot-assisted therapy is superior to other interventions to improve arm function (SMD fixed-effect model 0.21 [0.04; 0.38], *p*-value = 0.01) with better results among chronic patients (Bertani et al., 2017). Currently, robot-assisted therapy has level A evidence for treating chronic stroke phase according to the American Heart Association and American Stroke Association (Miller et al., 2010).

Although both interventions achieved relevant upper limb function results, CIMT has been considered a gold-standard treatment due to robust effects in the literature (Wolf et al., 2006; Thrane et al., 2014; Kwakkel et al., 2015). However, there is a lack of studies comparing CIMT and robot-assisted therapy to establish the specific benefits of each therapy approach on functional improvement and quality of life of chronic stroke patients. Preliminary discussions regarding motor recovery were released by our group at the *2018 Annals of Physical and Rehabilitation Medicine* (Terranova et al., 2018). The final results and discussion of this randomized clinical trial to demonstrate the difference between RT and CIMT regarding upper limb motor recovery and novel assessments on functionality and daily living activities of patients with chronic stroke are presented herein.

## Methods

This is a randomized clinical trial, with ethical approval under registration number 0961/11 (CAPPesq—*Comissão de Ética para Análise de Projetos de Pesquisa*) registered at ClinicalTrials.gov (ID number NCT02700061) and conducted at an outpatient physical rehabilitation site.

Patients were recruited at two different outpatient physical rehabilitation facilities of a national network reference Institute for physical medicine and rehabilitation care in São Paulo, Brazil.

Patients were required to be above 18 years of age, with clinical and radiological diagnoses (magnetic resonance imaging or computed tomography) of ischemic or hemorrhagic stroke of one or more events in the same brain hemisphere, time after stroke between six and 36 months after the date of the event. The participants should be clinically stable, according to neurological and cardiac evaluation. Upper limb impairments should be compatible with Brunnstrom's stages III and IV (Brunnstrom, 1966). Besides, participants were required to have at least a 20° wrist extension and at least 10° of metacarpophalangeal active extension, according to CIMT protocol. The individuals were required to repeat these movements at least three times in one min to ensure the constancy of their movements (Wolf et al., 2006; Thrane et al., 2014; Kwakkel et al., 2015).

Patients were not included if they presented muscle or bone injuries, disabling joint pain in upper limbs, Mini-Mental score below 20 points, psychological disturbances according to medical evaluation, or if they experienced previous treatment with robotic-assisted therapies. Treatment was discontinued if patients presented progressive worsening of spasticity according to Modified Ashworth Scale (Bohannon and Smith, 1987), a new stroke episode, or if they joined in another study protocol with upper limbs intervention.

After meeting these criteria, participants were invited to join in the study, and all the procedures, visits, intervention, and evaluation were explained. Then, they were requested to sign the Informed Consent Form and at last included in the study. After inclusion, they were randomly allocated to one of the intervention groups: 36 sessions of robot-assisted therapy (RT) or two weeks of Constraint-Induced Movement Therapy (CIMT), both of which combined with conventional treatment. Clinical measurements were carried out in four phases: before the beginning of the therapy (baseline), immediately after the end of the rehabilitation program, and follow-up assessments at three and 12 months after the end of their rehabilitation program.

### Intervention Group 1—Constraint-Induced Movement Therapy (CIMT)

In the CIMT group, patients received an outpatient conventional rehabilitation program for ten weeks, followed by two weeks (ten days) of CIMT, a total of 12 weeks of treatment. During the last two weeks, patients received 10 consecutive days (except weekends) of intensive therapies for six h per day at the Institution. Also, patients used a restraint mitt on the unaffected upper limb for 90% of their active day. Patients kept the restriction to perform the home activities during 14 consecutive days, including the weekend. On each day, therapists delivered different tasks for encouraging the use of the affected upper limb. CIMT protocol includes three fundamental components: shaping, task practice, and behavioral techniques. Shaping is a training method that requires the therapist's constant engagement to make the tasks gradually more challenging. Task practice is a repetitive practice of functional activity, and behavioral techniques include a package of strategies to transfer gains from the clinic to daily life (Uswatte et al., 2006).

Patients were encouraged to continuously perform functional activities according to a previous task selection when at home. Also, they were instructed to keep the training for the rest of the day and the weekend. Training included different tasks and activities related to daily living, such as to reach, grasp and lift different objects (cup, can, jar, bottle, basket, and others), to turn keys in locks, use light switches or knobs, to handle cutlery, to open and close drawers/doors, to dress up, to hold a phone or remote control, to clean a table and other tasks, all of which with the paretic limb (Wolf et al., 2006; Thrane et al., 2014; Kwakkel et al., 2015).

### Intervention Group 2—Robot-Assisted Therapy (RT)

Patients assigned to RT group performed the conventional institutional rehabilitation program, like the CIMT group; however, they concomitantly received three weekly sessions of 60 min RT for 12 weeks, composing 36 sessions. Patients used two robotic devices InMotion Arm® (shoulder, elbow movements, and grip training) and InMotion Wrist® (wrist movements). InMotion Robots are an interactive, anti-gravity system that supports the affected upper limb and allow guided exercises to reach targets located in different positions. This system enables intensive therapy, continuous feedback, adaptive training, task-specific training, and customized treatments. The 36 sessions were divided into nine sessions of shoulder and elbow training, nine sessions of wrist movements training, and nine sessions with a gripping device, and, in the last part, nine sessions of combined sessions with all devices in an integrated training. This robot-assisted protocol was based on previous protocols published in scientific literature (Lo et al., 2010).

### Conventional Rehabilitation Program

The conventional rehabilitation program was adjusted according to the patient's needs. It included weekly physical therapy sessions, occupational therapy, physical fitness, psychological therapy, speech therapy, nursing, and nutrition, and patients were also assisted by social service whenever prescribed. During conventional occupational therapy, patients received independence and safety training on basic activities of daily living and instrumental activities of everyday life, exercises to improve range and strength of lower limbs, sensory and tone stimulation, trunk stabilization, and gait training.

Our primary outcome is the improvement of proximal and distal upper-limb motor control and upper-limb motor recovery measured by Wolf Motor Function Test time (WMFT time) and Fugl-Meyer Assessment—Upper Limb (FMA-UL), respectively. The WMFT measures 17 timed functional tasks with a score that ranges from 0 to 120 s, with higher scores indicating worse functioning (Pereira et al., 2011), whereas FMA-UL is a scale whose score ranges from 0 to 66 points, in which higher scores indicate better functioning (Fugl-Meyer et al., 1975). Extending the preliminary publication at the *2018 Annals of Physical and Rehabilitation Medicine* (Terranova et al., 2018), other assessments were applied to explore the effects of both techniques. Such assessments measured functional and motor recovery with the scales Wolf Motor Functional Test (Ability domain), Arm Motor Ability Test (AMAT), Functional Independence Measure (MIF), and Modified Ashworth Scale (MAS). Also, the Stroke Impact Scale (SIS) in the domains of strength, activities of daily living, and hand function were used to assess activities of daily living (Hatem et al., 2016).

Trained raters collected data according to the instructions of each measurement. After standardization, they evaluated the patients in four different phases: at baseline (before the intervention), at the end of the rehabilitation program.

As registered at ClinicalTrials.gov, evaluators tested the patients' neuronal activity with transcranial magnetic stimulation and electroencephalography. These evaluations were also conducted three and 12 months after the rehabilitation program for follow-up. Such assessments will be subject to future publication due to their different research question and objectives.

There was an initial estimation of 62 patients in each treatment group, a total sample size of 124 stroke patients. The sample size was estimated according to Wolf et al. (2006) in which they found a reduction of 26% in time to perform the tasks proposed in WMFT assessment after a CIMT rehabilitation program. We defined the power of 80% and alpha of 0.05, bicaudal.

The study was terminated after three years of recruitment, with 51 participants enrolled, randomized, and allocated into one of both intervention groups.

Randomization was made by a computer program that generated blocks of four, six, and eight at a ratio of 1:1. After the randomization sequence was generated, a clinical research analyst kept the numbers in sealed opaque envelopes. After the patient signed the informed consent form and the baseline assessments, they were allocated into one of both intervention groups according to the random allocation sequence described within the envelope. The allocation sequence was concealed from the investigator.

### Statistical Methods

Statistical analysis was performed with STATA (StataCorp LLC, Texas, USA). To analyze intervention group Intra and inter-differences, non-parametric tests were applied to test the change in score after RT and CIMT sessions, given the results of normality tests. *Post-hoc* analyses were carried out on the overall treatment effects regardless of the treatment groups, and all demographic characteristics were tested for confounders; this statistical development addressed the concern that one or more demographic variable could be the cause of our findings, an issue that was not considered in the preliminary release (Terranova et al., 2018). Finally, a *post-hoc* futility analysis was conducted to disclose the impact of this trial's early termination. The significance level was set at *p* < 0.05 for all statistical analyses.

## Results

**Figure 1:**
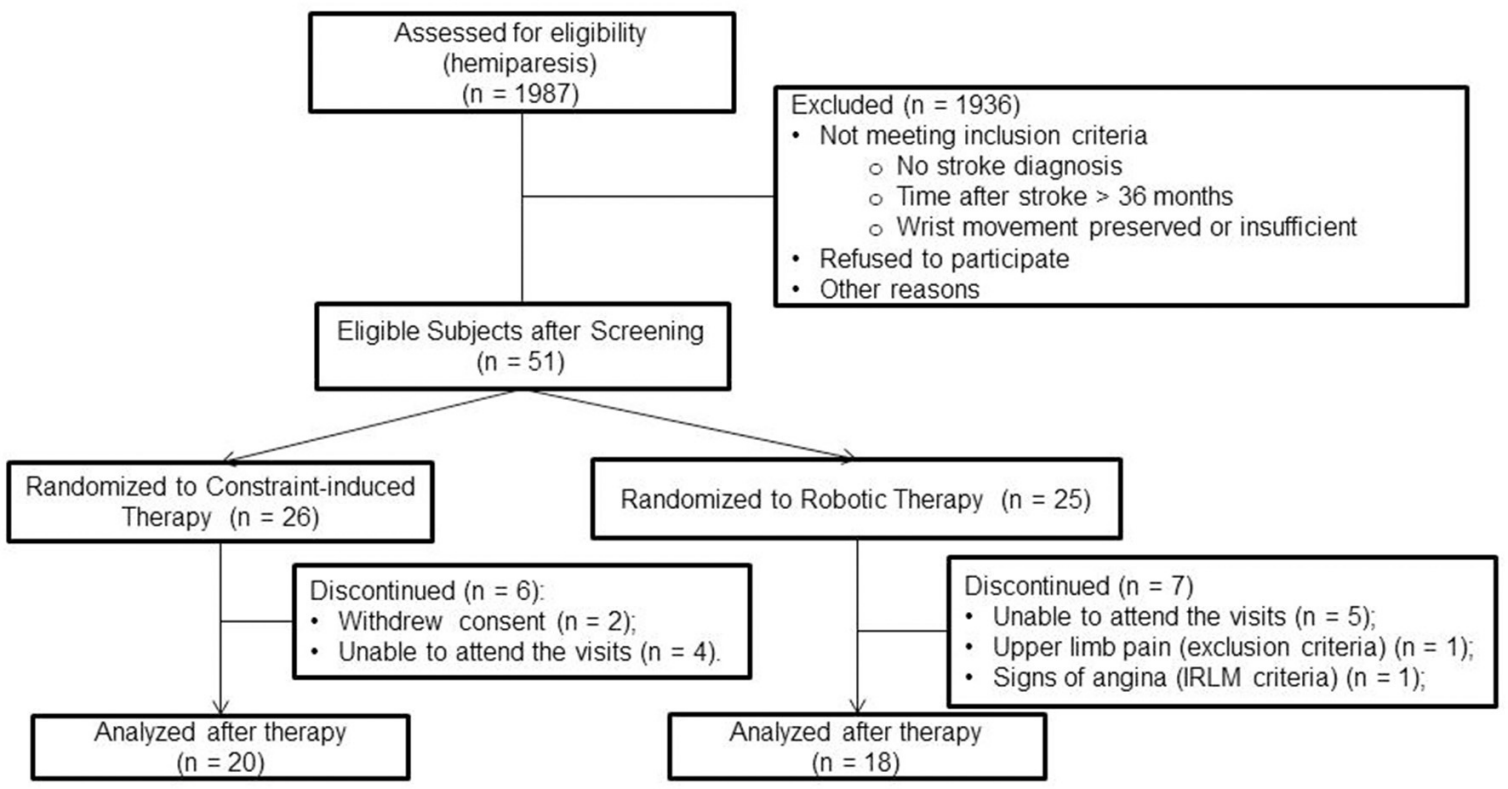
Inclusion, treatment, and analysis flow diagram.

From May 2012 to May 2015, associates contacted 1.272 patients. Eighty-seven of them fulfilled the eligibility criteria, and 51 were enrolled. Thirteen patients dropped out. In the CIMT group, two patients withdrew consent, and four were unable to attend the treatment visits. In contrast, five patients could not attend the treatment visits in the robotic therapy group. Two others presented discontinuation criteria, pain in the upper limb, and medical instability (described in Figure 1). Patient characteristics at baseline were relatively well-balanced between groups (Table 1).

**Table 1:** Demographic and baseline characteristics.

	**CIMT**	**Robotic Therapy**
Patients, N	26	25
Women, N (%)	15 (57.7)	10 (66.67)
Age, mean years (SD)	59.35 (16.48)	60.72 (12.38)
Time after stroke, mean months (SD)	18.96 (9.68)	14.32 (7.53)
Plegic side, right (%)	16 (61.54)	11 (44)
WMFT^‡^	95.63 (66.59−223.49)	93.6 (76.32−468.21)
Fugl Meyer^‡^	52.5 (46.75−57.0)	51.0 (42.5−56.5)
AMAT (Ability)^‡^	3.52 (2.72−4.62)	3.34 (2.65−4.05)
AMAT (Quality)^‡^	2.97 (2.34−3.69)	2.85 (2.15−3.55)
MAS^‡^	0.17 (0−0.50)	0.17 (0−0.50)
FIM^‡^	78 (72−80)	76 (70−79)
SIS total score^‡^	69.39 (59.09−74.23)	64.51 (51.31−68.82)
SIS strength^‡^	65.62 (50−75)	43.75 (37.5−62.5)
SIS Activities of Daily Living^‡^	67.5 (50−80)	61.25 (47.5−72.5)
SIS Hand Function^‡^	40 (10−55)	17.5 (0−37.5)

*CIMT, constraint-induced movement therapy; SD, standard deviation; WMFT, Wolf Motor Function Test; AMAT, Arm Motor Ability Test; MAS, Modified Ashworth Scale; FIM, Functional Independence Measure; SIS, Stroke Impact Scale*.

‡ *Median, interquartile ranges were used due to non-parametric distribution*.

The normality test (Shapiro-Wilk) showed that the variables of the primary outcome (WMFT and FMA-UL) could not be considered parametrical, therefore statistical analyses were conducted with Wilcoxon and Mann-Whitney tests. Considering the overall treatment analysis, regardless of the intervention group, a significant improvement for WMFT and FMA-UL and similar results were found for most secondary outcomes (Table 2). There was a slight but not statistically significant difference between groups (Table 3) regarding the primary outcomes. The mean change, measured by FMA-UL, was 4.5 points for CIMT and 2.7 points for RT (*p* = 0.187). Regarding WMFT, the improvement was −24.36 and −11.09 s for CIMT and RT, respectively (*p* = 0.293). Other assessments were consistent with the primary outcome results as no significant differences were found between both groups (Table 3). No confounder was found among the demographical characteristics, therefore no statistical adjustments were applied.

**Table 2:** Overall treatment analysis.

**Assessment**	**Before the treatment^‡^**	**After the treatment^‡^**	***p*-value**
WMFT, median time, seconds	93.6 (67.76−316.9)	76.96 (50.02−197,98)	**0.011**
FMA—UL	51 (45−57)	56.5 (49−62)	**<0.001**
WMFT (Ability), score	3.6 (2.8−3.87)	3.93 (3.26−4.47)	**<0.001**
AMAT (Ability), score	3.5 (2.69−4)	3.81 (3.24−4.65)	**<0.001**
AMAT (Quality), score	2.97 (2.24−3.65)	3.55 (2.86−4.10)	**<0.001**
MAS, score	0.17 (0−0.5)	0.11 (0−0.28)	**0.005**
FIM, score	77 (71−80)	79.5 (78−81)	**<0.001**
SIS (total score)	65.32 (55.79−72.34)	73.56 (62.83−79.31)	**<0.001**
SIS strength	56.25 (37.5−75)	62.5 (50−75)	0.075
SIS Activities of Daily Living	63.75 (47.5−77.5)	75 (60−82.5)	**<0.001**
SIS Hand Function	30 (5−45)	55 (35−65)	**<0.001**

*CIMT and RT combined*.

*CMIT, constraint-induced movement therapy; CI95%, 95% confidence interval; WMFT, Wolf Motor Function Test; FMA—UL, Upper Limb Fugl Meyer Assessment; AMAT, Arm Motor Ability Test; MAS, Modified Ashworth Scale; FIM, Functional Independence Measure; SIS, Stroke Impact Scale;*

‡ *Median, interquartile range*.

*The bold values are the statistically significant p-values*.

**Table 3:** Treatment group analysis.

**Treatment group analysis**	**CIMT^‡^**	**Robotic Therapy^‡^**	**p-value**
WMFT median time, seconds	−24.36 (−133.80−5.08)	−11.09 (−27.56−8.87)	0.293
FMA—UL	4.5 (1.5−7)	2.7 (−2−6)	0.187
WMFT (Ability)	0.27 (0.1−0.7)	0.30 (0.27−0.6)	0.769
AMAT (Ability)	0.27 (0.19−0.91)	0.41 (0.17−0.79)	0.988
AMAT (Quality)	0.50 (0.22−0.79)	0.53 (0.17−0.89)	0.792
MAS	−0.05 (−0.66−0)	0 (−0.11−0.05)	0.014
FIM	1.5 (0−5.25)	3.0 (2.0−5.75)	0.517
SIS (total score)	6.77 (0−15.20)	7.04 (1.89−11.64)	0.761
SIS strength	0 (−12.5−18.75)	15.63 (−6.25−25)	0.122
SIS Activities of Daily Living	10 (0−15)	10.0 (−2.5−17.5)	0.855
SIS Hand Function	15.0 (5−25)	17.5 (5.0−25)	0.939

*CMIT, constraint-induced movement therapy; WMFT, wolf motor function test (mean time in seconds); FMA—UL, upper limb fugl meyer assessment; AMAT, arm motor ability test; MAS, modified ashworth scale; FIM, functional independence measure; SIS, stroke impact scale;*

‡ *Median change from baseline*.

### *Post-hoc* Futility Analysis

Based on the differences of clinical improvement between both groups measured by WMFT, the futility analysis evidenced that a statistically significant difference (*p*-value < 0.05 with the power of 80% or above) would be reached with the inclusion of 144 participants per group if the results of each patient maintained the same standard. However, the effect size would still be mild (rank biserial correlation, *r* = 0.12).

## Discussion

As evidenced by previous studies, both CIMT and RT improved upper limb motor function and daily living activities of patients with stroke sequelae. Subtle differences between both techniques were found for all outcomes. Nonetheless, they were not statistically significant. This finding was shown in the preliminary results of our study presented at the *2018 Annals of Physical and Rehabilitation Medicine* (Terranova et al., 2018), as both groups improved motor recovery, but no differences were found between both techniques (*p*-values 0.43 and 0.19 for WMFT and FMA, respectively). As evidenced in the results herein discussed, secondary outcomes showed that motor recovery was followed by improvements in strength, spasticity, and independence. These improvements suggest that the benefits of the physical rehabilitation program were robust regardless of the intervention group.

A publication from the Cochrane Database of Systematic Reviews concluded that CIMT shows limited upper limb motor function improvements, not necessarily representing a more significant reduction in disability than conventional therapies (Corbetta et al., 2015). However, another meta-analysis evidenced that CIMT can be superior to conventional rehabilitation (Thrane et al., 2014), regardless of the time since stroke. The meta-analysis addressed daily living activities and function and showed that CIMT improved both domains. Conversely, the Cochrane review discusses that, for patients in the chronic stage of stroke, CIMT seems to improve disability by adaptation strategies for daily living activities (ADL) without necessarily reducing disability. This conclusion appears to agree with other authors who discuss that CIMT shows more significant effects on motor function recovery in earlier stages of stroke, as the restitution of neurological functions is more likely to occure (Wolf et al., 2006; Thrane et al., 2014; Kwakkel et al., 2015). Oppositely, our findings have shown otherwise, as our participants, with an average time after stroke of 14.32 and 18.96 months in the CIMT and RT groups, respectively, improved motor function recovery as well as the other relevant aspects of strength, spasticity, and independence, even though neither CIMT nor RT is explicitly applied for these last three domains (Kahn et al., 2006; Lo et al., 2010; Corbetta et al., 2015). This is an important finding as it suggests that, even during the chronic phase of stroke, the physical rehabilitation program yields benefits to patients with stroke.

A critical aspect of CIMT is its applicability. The execution of CIMT requires one dedicated therapist for three to six hours per day, for 10 or even 15 consecutive days (Thrane et al., 2014) to ensure the performance and repetition of the tasks with the patient. Moreover, it is essential to evaluate the patients' cognitive and emotional aspects and the family support throughout the CIMT protocol and the therapists' availability before prescribing this rehabilitation technique. On the other hand, CIMT may be advantageous for inpatient facilities, where patients may be treated in a more intense program for a shorter period.

Regarding Robot-Assisted Therapy, randomized clinical trials that applied this technique have shown upper limb motor improvements without, however, evident superiority when compared to intensive training (Kahn et al., 2006; Volpe et al., 2008; Lo et al., 2010). Nonetheless, the specialized literature is still evolving. Once other publications on clinical trials for patients with stroke during acute and chronic phases (Fasoli et al., 2003; Ferraro et al., 2003; Prange et al., 2006; Masiero et al., 2007), Robot-Assisted Therapy can be recommended for inpatient or outpatient facilities. It offers the amount of motor practice needed to relearn upper limb motor skills (Miller et al., 2010).

Another important aspect is the cost-effectiveness of RT over CIMT. It is estimated that RT could reduce the upper limb physical rehabilitation costs by optimizing the therapist's time and providing a high number of specific movement repetitions (about 1.000 repetitions per session). Lo et al. (2010) demonstrated an economic analysis comparing robot devices and conventional therapies, showing that robots can reduce patients' physical rehabilitation costs after stroke (Lo et al., 2010). Even though any cost analysis must be validated for different countries, researchers estimate that CIMT increases the cost of conventional rehabilitation program (Light, 2015). Moreover, some of the robotic systems generate an assessment of patients' kinematic metrics related to motor function used as a measurement, which is another advantage compared to CIMT (Bosecker et al., 2010). As the robotic system automatically drives the tasks regardless of a therapist's extensive presence, in our study, we managed to standardize and optimize the 60 min sessions three times a week.

Another aspect that should be observed is the applicability of RT. Recent studies indicate positive results regarding the usability and feasibility of the robotic devices compared to other interventions, showing that patients report greater satisfaction and motivation when assigned to a robotic device during the rehabilitation process (Masiero et al., 2007; Hughes et al., 2011; Park et al., 2013; Lledó et al., 2016; Lee et al., 2017; Resquín et al., 2017). Greater satisfaction may be related to higher adherence. Nonetheless, this was not observed in our study. The number of dropouts along the interventions was similar and not necessarily due to the interventions themselves, even though we believe the shorter RT session duration may also be considered an advantage CIMT regarding adherence. Although we did not observe a difference in adherence, other authors observed more significant impacts in the patients' degree of acceptance and compliance with the rehabilitation treatment due to technology use (Bovolenta et al., 2009, 2011). Such acceptance may be explained by the difference in routine between both treatments. The RT demands a 1-h session three times a week, whereas the CIMT approach holds the patients active for about 90% (Bovolenta et al., 2009, 2011; Light, 2015). In our study, the professionals reported that the patients of the CIMT group manifested tiredness and discomfort. Oppositely, the patients of the RT group had better engagement and performance during the sessions. Nonetheless, such events could not be measured, and the final results did not evidence significant differences between the interventions.

According to previous studies, CIMT and RT have different indications, as CIMT is suggested for patients with potential for wrist and finger control and RT for shoulder and elbow motor recovery (Wolf et al., 2006; Lo et al., 2010). Moreover, RT has a greater potential to recover remaining motor components such as stretching, mobility, strength, tonus, range of motion, and smoothness (Fasoli et al., 2003; Ferraro et al., 2003; Kahn et al., 2006; Prange et al., 2006; Masiero et al., 2007; Volpe et al., 2008; Lo et al., 2010). CIMT protocol includes task-specific training with the affected upper limb to improve the time and ability to perform tasks related to the ADL, such as eating, drinking, reaching, and carrying objects (Wolf et al., 2006). Therefore, it is suggested that CIMT and RT could be complementary interventions during the rehabilitation process. In our results, secondary outcomes evidenced that RT was not necessarily inferior to CIMT on task-specific training, as the values of AMAT and WMFT for both ability and quality of motor function for specific tasks of daily activities were statistically similar regardless of the intervention group. Considering that the participants were in the chronic phase of stroke sequelae, combining both techniques seems promising.

The combination of the CIMT and the RT has already been discussed previously in the literature. Two studies showed that RT combined with CIMT led to better improvements in upper limb functional ability (*p* = 0.01), measured by WMFT, to perform motor tasks when compared to RT combined with conventional therapy or RT alone. Therefore, CIMT could produce more significant improvements when applied in Robot-Assisted Therapy once CIMT would reinforce adaptation strategies after the patient acquired the maximum potential motor in the RT sessions (Hsieh et al., 2014, 2016).

There are significant differences regarding the indications and execution of CIMT or RT. The range of patients able to perform CIMT protocol is limited due to restricting criteria such as the minimum of 10° of wrist extension and 10° abduction/extension of the thumb and at least a 10° extension in at least two other fingers. Indeed, this was an important reason for the low percentage (about 5%) of patients who met our study's inclusion criteria. It is estimated that only 6.5% of stroke patients can receive CIMT (Fabbrini et al., 2014). This criterium was the main reason for our study's early termination with 51 patients included, about 41% of the expected sample size, and may also partially explain the patients' improvement, once, even though they were in the chronic phase of stroke, they had some level of independence, as the baseline FIM was 78 and 76 for CIMT and RT, respectively. Hence, therapists should consider the flexibility for prescribing robot-assisted therapy another advantage once these devices can be adjustable to different disability degrees. They offer adaptation and graduation of tasks, contemplating a larger disability spectrum.

As observed in previous studies, valuable and motivational robotic systems are designed to increase patient adherence to treatment once the patient's motivation and engagement need to be stimulated to achieve better rehabilitation outcomes during the rehabilitation programs (Grahn et al., 2000; Resquín et al., 2017). This shows that even though robotic therapy has yet to deliver better outcomes than CIMT, our results and the specialized literature support the use of robotic devices during rehabilitation programs for stroke patients.

Even though our results should consider the small effect sizes and the possible lack of power to detect subtle differences, the agreement between motor recovery and function [FMA-UL and WMFT, respectively (Fugl-Meyer et al., 1975; Pereira et al., 2011)] and the secondary outcomes of function, independence, spasticity, and strength suggests our results may be considered robust. We also acknowledge that our protocol compared 36 sessions of RT with 10 days of CIMT protocol, with a total time of 18 h of RT distributed along 12 weeks and 60 h of CIMT concentrated in two weeks only, which may jeopardize comparability. Nonetheless, we understand comparability is still possible once the RT or CIMT protocols were combined with a conventional rehabilitation program, as stated in the methods. As for the influence of the number of sessions and time, even though both interventions are validated protocols, it is not possible to isolate the effects of both variables on our patients' recovery, as they may have favored one technique over the other or even be the sole cause of the differences we found between them. Therefore, future study designs on the comparison of RT and CIMT should analyze the influence of time and number of sessions over the results and consider the inclusion of other assessments along with both protocols.

Future studies should expand the knowledge on both techniques. Regardless of the interventions herein discussed, the best responders for each one of them are not entirely established. Understanding this matter could provide health professionals with the best strategy to rehabilitate their patients. Also, the combination of both RT and CIMT demands proper investigation. It could deliver the best of both strategies and reduce their disadvantages once the patient would not be subject to only one type of therapy. Concerning disadvantages, the costs of both interventions need special attention, and future studies in this regard may be decisive for the choice of one technique over the other. At last, the long-term effects of both techniques should also be researched.

Our study showed that chronic stroke patients improved upper limb function, motor recovery, and daily living activities after robotic-assisted therapies (RT) or Constraint-Induced Motor Therapy (CIMT), both combined with conventional occupational therapy RT is not different from CIMT. The prescription for a rehabilitation program should consider the RT advantages over CIMT, however, long-term effects and treatment adherence to robotic-assisted therapy are yet to be studied in the future. At last, we consider that future studies should test the combination of both CIMT and RT techniques and the long-term effects of robotic therapy.

## Data Availability Statement

The datasets presented in this article are not readily available. A previous specific approval by an Ethics Committee is required, according to Brazilian regulations. Requests to access the datasets should be directed to Artur César Aquino Santos, artur.santos@hc.fm.usp.br.

## Ethics Statement

The studies involving human participants were reviewed and approved by CAPPesq—Comissão de Ética para Análise de Projetos de Pesquisa. The patients/participants provided their written informed consent to participate in this study.

## Author Contributions

TT: concepts, design, definition of intellectual content, literature search, data acquisition, data analysis, statistical analysis, manuscript preparation, and manuscript editing. MS: definition of intellectual content, literature search, data acquisition, data analysis, statistical analysis, manuscript preparation, and manuscript editing. AS: data acquisition, data analysis, statistical analysis, and manuscript preparation. FA: concepts, design, definition of intellectual content, literature search, data analysis, and manuscript preparation. MI: concepts, design, definition of intellectual content, literature search, data acquisition, manuscript editing, and manuscript review. FF: concepts, design, definition of intellectual content, data analysis, manuscript preparation, and manuscript review. LB: concepts, design, definition of intellectual content, literature search, data analysis, manuscript review, and guarantor. All authors contributed to the article and approved the submitted version.

## Conflict of Interest

The authors declare that the research was conducted in the absence of any commercial or financial relationships that could be construed as a potential conflict of interest.

## References

[B1] Barreca S. Wolf S. L. Fasoli S. Bohannon R. (2003). Treatment interventions for the paretic upper limb of stroke survivors: a critical review. Neurorehabil. Neural Repair 17, 220–226. 10.1177/0888439003259415 14677218

[B2] Bertani R. Melegari C. De Cola M. C. Bramanti A. Bramanti P. Calabrò R. S. (2017). Effects of robot-assisted upper limb rehabilitation in stroke patients: a systematic review with meta-analysis. Neurol. Sci. 38, 1561–1569. 10.1007/s10072-017-2995-5 28540536

[B3] Bohannon R. W. Smith M. B. (1987). Interrater reliability of a modified Ashworth scale of muscle spasticity. Phys. Ther. 67, 206–207. 10.1093/ptj/67.2.206 3809245

[B4] Bosecker C. Dipietro L. Volpe B. Krebs H. I. (2010). Kinematic robot-based evaluation scales and clinical counterparts to measure upper limb motor performance in patients with chronic stroke. Neurorehabil. Neural Repair 24, 62–69. 10.1177/1545968309343214 19684304 PMC4687968

[B5] Bovolenta F. Goldoni M. Clerici P. Agosti M. Franceschini M. (2009). Robot therapy for functional recovery of the upper limbs: a pilot study on patients after stroke. J. Rehabil. Med. 41, 971–975. 10.2340/16501977-0402 19841826

[B6] Bovolenta F. Sale P. Dall'Armi V. Clerici P. Franceschini M. (2011). Robot-aided therapy for upper limbs in patients with stroke-related lesions. A brief report of a clinical experience. J. Neuroeng. Rehabil. 8:18. 10.1186/1743-0003-8-18 21477331 PMC3086823

[B7] Brunnstrom S. (1966). Motor testing procedures in hemiplegia: based on sequential recovery stages. Phys. Ther. 46, 357–375. 10.1093/ptj/46.4.357 5907254

[B8] Corbetta D. Sirtori V. Castellini G. Moja L. Gatti R. (2015). Constraint-induced movement therapy for upper extremities in people with stroke. Cochrane Database Syst. Rev. 2015:CD004433. 10.1002/14651858.CD004433.pub3 26446577 PMC6465192

[B9] Dettmers C. Teske U. hamzei F. Uswatte G. Taub E. Weiller C. (2005). Distributed form of constraint-induced movement therapy improves functional outcome and quality of life after stroke. Phys. Med. Rehabil. 86, 204–209. 10.1016/j.apmr.2004.05.007 15706544

[B10] Dipietro L. Ferraro M. Palazzolo J. J. Krebs H. I. Volpe B. T. Hogan N. (2005). Customized interactive robotic treatment for stroke: EMG-triggered therapy. IEEE Trans. Neural Syst. Rehabil. Eng. 13, 325–334. 10.1109/TNSRE.2005.850423 16200756 PMC2752646

[B11] Dipietro L. Krebs H. I. Volpe B. T. Stein J. Bever C. Mernoff S. T.. (2012). Learning, not adaptation, characterizes stroke motor recovery: evidence from kinematic changes induced by robot-assisted therapy in trained and untrained task in the same workspace. IEEE Trans. Neural Syst. Rehabil. Eng. 20, 48–57. 10.1109/TNSRE.2011.2175008 22186963 PMC4687974

[B12] Fabbrini S. Casati G. Bonaiuti D. (2014). Is CIMT a rehabilitative practice for everyone? predictive factors and feasibility. Eur. J. Phys. Rehabil. Med. 50, 505–514. 24704938

[B13] Fasoli S. E. Krebs H. I. Stein J. Frontera W. R. Hogan N. (2003). Effects of robotic therapy on motor impairment and recovery in chronic stroke. Arch. Phys. Med. Rehabil. 84, 477–482. 10.1053/apmr.2003.50110 12690583

[B14] Ferraro M. Palazzolo J. J. Krol J. Krebs H. I. Hogan N. Volpe B. T. (2003). Robot-aided sensorimotor arm training improves outcome in patients with chronic stroke. Neurology 61, 1604–1607. 10.1212/01.WNL.0000095963.00970.68 14663051

[B15] Fugl-Meyer A. R. Jääskö L. Leyman I. Olsson S. Steglind S. (1975). The post-stroke hemiplegic patient. A method for evaluation of physical performance. Scand. J. Rehabil. Med. 7, 13–31. 1135616

[B16] Grahn B. Ekdahl C. Borgquist L. (2000). Motivation as a predictor of changes in quality of life and working ability in multidisciplinary rehabilitation. A two-year follow-up of a prospective controlled study in patients with prolonged musculoskeletal disorders. Disabil. Rehabil. 22, 639–654. 10.1080/096382800445443 11087060

[B17] Hatem S. M. Saussez G. Della Faille M.. (2016). Rehabilitation of motor function after stroke: a multiple systematic review focused on techniques to stimulate upper extremity recovery. Front. Hum. Neurosci. 10:442. 10.3389/fnhum.2016.00442 27679565 PMC5020059

[B18] Hesse S. Schimdt H. Werner C. Bardeleben A. (2003). Upper and lower extremity robotics devices for rehabilitation and for studing motor control. Curr. Opin. Neurol. 16, 705–710. 10.1097/00019052-200312000-00010 14624080

[B19] Hsieh Y. Liing R. J. Lin K. C. Wu C. Y. Liou T. H. Lin J. C.. (2016). Sequencing bilateral robot-assisted arm therapy and constraint-induced therapy improves reach to press and trunk kinematics in patients with stroke. J. Neuroeng. Rehabil. 22:31. 10.1186/s12984-016-0138-5 27000446 PMC4802889

[B20] Hsieh Y. Lin K. C. Horng Y. S. Wu C. Y. Wu T. C. Ku F. L. (2014). Sequential combination of robot-assisted therapy and constraint-induced therapy in stroke rehabilitation: a randomized controlled trial. J. Neurol. 261, 1037–1045. 10.1007/s00415-014-7345-4 24748465

[B21] Hughes A. M. Burridge J. Freeman C. T. Donnovan-Hall M. Chappell P. H. Lewin P. L.. (2011). Stroke participants' perceptions of robotic and electrical stimulation therapy: a new approach. Disabil. Rehabil. Assist. Technol. 6, 130–138. 10.3109/17483107.2010.509882 20698789

[B22] Johnson W. Onuma O. Owolabi M. Sachdev S. (2016). Stroke: a global response is needed [editorial]. Bull. World Health Organ. 94, 634–634A. 10.2471/BLT.16.181636 27708464 PMC5034645

[B23] Kahn L. E. Zygman M. L. Rymer W. Z. Reinkensmeyer D. J. (2006). Robot-assisted reaching exercise promotes arm movement recovery in chronic hemiparetic stroke a randomized controlled pilot study. J. Neuroeng. Rehabil. 3:12. 10.1186/1743-0003-3-12 16790067 PMC1550245

[B24] Krebs H. I. Dipietro L. Levy-Tzedek S. (2008). A paradigm shift for rehabilitation robotics. IEEE-EMBS Magazine 27, 61–70. 10.1109/MEMB.2008.919498

[B25] Krebs H. I. Hogan N. (2006). Therapeutic robotics: a technology push: stroke rehabilitation is being aided by robots that guide movement of shoulders and elbows, wrists, hands, arms and ankles to significantly improve recovery of patients. Proc. IEEE Spec. Issue Med. Robot. 94, 1727–1738. 10.1109/JPROC.2006.880721 19779587 PMC2749278

[B26] Kunkel A. Kopp B. Muller G. Villringer K. Villringer A. Taub E.. (1999). Constraint-induced movement therapy for motor recovery in chronic stroke patients. Phys. Med. Rehabil. 80, 624–628. 10.1016/S0003-9993(99)90163-6 10378486

[B27] Kwakkel G. Veerbeek J. M. van Wegen E. E. Wolf S. L. (2015). Constraint-induced movement therapy after stroke. Lancet Neurol. 14, 224–234. 10.1016/S1474-4422(14)70160-7 25772900 PMC4361809

[B28] Langhorne P. Bernhardt J. Kwakkel G. (2011). Stroke rehabilitation. Lancet 377, 1693–1702. 10.1016/S0140-6736(11)60325-5 21571152

[B29] Lee K. W. Kim S. B. Lee J. H. Lee S. J. Kim J. W. (2017). Effect of robot-assisted game training on upper extremity function in stroke patients. Ann. Rehabil. Med. 41, 539–546. 10.5535/arm.2017.41.4.539 28971037 PMC5608660

[B30] Levin M. F. Kleim J. A. Wolf S. L. (2009). What do motor “recovery” and “compensation” mean in patients following stroke? Neurorehabil. Neural Repair. 23, 313–319. 10.1177/1545968308328727 19118128

[B31] Light K. (2015). Constraint-induced movement therapy: home-training is beneficial and cost-effective. Physiotherapy 101, e873–e874. 10.1016/j.physio.2015.03.1702

[B32] Lledó L. D. Díez J. A. Bertomeu-Motos A. Ezquerro S. Badesa F. J. Sabater-Navarro J. M.. (2016). A comparative analysis of 2D and 3D tasks for virtual reality therapies based on robotic-assisted neurorehabilitation for post-stroke patients. Front. Aging Neurosci. 8:205. 10.3389/fnagi.2016.00205 27616992 PMC4999455

[B33] Lo A. C. Guarino P. D. Richards L. G. Haselkorn J. K. Wittenberg G. F. Federman D. G.. (2010). Robot-assisted therapy for long-term upper-limb impairment after stroke. N. Engl. J. Med. 362, 1772–1783. 10.1056/NEJMoa0911341 20400552 PMC5592692

[B34] Masiero S. Celia A. Rosati G. Armani M. (2007). Robotic-assisted rehabilitation of the upper limb after acute stroke. Arch. Phys. Med. Rehabil. 88, 142–149. 10.1016/j.apmr.2006.10.032 17270510

[B35] Miller E. L. Murray L. Richards L. Zorowitz R. D. Bakas T. Clark P.. (2010). American heart association council on cardiovascular nursing and the stroke council. Comprehensive overview of nursing and interdisciplinary rehabilitation of the stroke patient: a scientific statement from the American heart association. Stroke 41, 2402–2448. 10.1161/STR.0b013e3181e7512b 20813995

[B36] Morris D. M. Taub E. Mark V. W. (2006). Constraint-induced movement therapy: characterizing the intervention protocol. Eura Medicophys. 42, 257–268. 17039224

[B37] Nichols-Larsen D. S. Clark P. C. Zeringue A. Greenspan A. Blanton S. (2005). Factors influencing stroke survivors' quality of life during subacute recovery. Stroke. 36, 1480–1484. 10.1161/01.STR.0000170706.13595.4f 15947263

[B38] Page S. J. Levine P. (2007). Modified constraint-induced movement therapy extension: using remote technologies to improve function. Arch. Phys. Med. Rehabil. 88, 922–927. 10.1016/j.apmr.2007.03.038 17601475

[B39] Park W. Jeong W. Kwon G. H. Kim Y. H. Kim L. (2013). A rehabilitation device to improve the hand grasp function of stroke patients using a patient-driven approach. IEEE Int. Conf. Rehabil. Robot. 2013:6650482. 10.1109/ICORR.2013.6650482 24187299

[B40] Pereira N. D. Michaelsen S. M. Menezes I. S. Ovando A. C. Lima R. C. M. Salmela L. F. (2011). Confiabilidade da versão brasileira do Wolf Motor Function Test em adultos com hemiparesia. Rev. Bras Fisioter 15, 257–265. 10.1590/S1413-35552011000300013 21829991

[B41] Prange G. B. Jannink M. J. Groothuis-Oudshoorn C. G. Hermens H. J. Ijzerman M. J. (2006). Systematic review of the effect of robot-aided therapy on recovery of the hemiparetic arm after stroke. J. Rehabil. Res. Dev. 43, 171–184. 10.1682/JRRD.2005.04.0076 16847784

[B42] Resquín F. Gonzalez-Vargas J. Ibáñez J. Brunetti F. Dimbwadyo I. Carrasco L.. (2017). Adaptive hybrid robotic system for rehabilitation of reaching movement after a brain injury: a usability study. J. Neuroeng. Rehabil. 14:104. 10.1186/s12984-017-0312-4 29025427 PMC5639749

[B43] Taub E. Miller N. E. Novack T. A. Cook E. W. Fleming W. C. Nepomuceno C. S.. (1993). Technique to improve chronic motor defict after stroke. Arch. Phys. Med. Rehabil. 74, 347–354. 8466415

[B44] Taub E. Uswatte G. King D. K. Morris D. Crago J. E. Chatterjee A. (2006). A placebo-controlled trial of constraintinduced movement therapy for upper extremity after stroke. Stroke 37, 1045–1049. 10.1161/01.STR.0000206463.66461.97 16514097

[B45] Terranova T. Simis M. Santos A. Imamura M. Alfieri F. Fregni F.. (2018). Comparing effects of constraint-induced movement therapy and robotic therapy: randomized clinical trial. Ann. Phys. Rehabil. Med. 61:e34. 10.1016/j.rehab.2018.05.076

[B46] Thrane G. Friborg O. Anke A. Indredavik B. (2014). A meta-analysis of constraint-induced movement therapy after stroke. J. Rehabil. Med. 46, 833–842. 10.2340/16501977-1859 25182341

[B47] Uswatte G. Taub E. Morris D. Barman J. Crago J. (2006). Contribution of the shaping and restraint components of constraint-induced movement therapy to treatment outcome. NeuroRehabilitation 21, 147–156. 10.3233/NRE-2006-21206 16917161

[B48] Volpe B. T. Lynch D. Rykman-Berland A. Galgano M. Hogan N. Krebs H.. (2008). Intensive sensorimotor arm training mediated by therapist or robot improves hemiparesis in patients with chronic stroke. Neurorehabil. Neural Repair 22, 305–310. 10.1177/1545968307311102 18184932 PMC4943019

[B49] Wolf S. Blanton S. Baer H. Breshears J. Butler A. J. (2002). Repetitive task practice: a critical review of constraintinduced movement therapy in stroke. Neurologist 8, 325–338. 10.1097/00127893-200211000-00001 12801434 PMC3572508

[B50] Wolf S. L. Lecraw D. E. Barton L. A. Jann B. B. (1989). Forced use of hemiplegic upper extremities to reverse the effect of learned nonuse among chronic stroke and head-injured patients. Exp. Neurol. 104, 125–132. 10.1016/S0014-4886(89)80005-6 2707361

[B51] Wolf S. L. Winstein C. J. Miller J. P. Taub E. Uswatte G. Morris D.. (2006). Effect of constraint-induced movement therapy on upper extremity function 3 to 9 months after stroke: the EXCITE randomized clinical trial. JAMA 296, 2095–2104. 10.1001/jama.296.17.2095 17077374

